# Huangbai Liniment Ameliorates Skin Inflammation in Atopic Dermatitis

**DOI:** 10.3389/fphar.2021.726035

**Published:** 2021-08-31

**Authors:** Ting Zheng, Miao Fan, Yunbo Wei, Jinhong Feng, Pengcheng Zhou, Xin Sun, Anqi Xue, Cheng Xue Qin, Di Yu

**Affiliations:** ^1^ Shandong Analysis and Test Center, Laboratory of Immunology for Environment and Health, Qilu University of Technology (Shandong Academy of Sciences), Jinan, China; ^2^ School of Pharmaceutical Sciences, Qilu University of Technology (Shandong Academy of Sciences), Jinan, China; ^3^ School of Pharmaceutical Science, Shandong University, Jinan, China; ^4^ The University of Queensland Diamantina Institute, Translational Research Institute, Brisbane, QLD, Australia; ^5^ School of Food Science and Engineering, Qilu University of Technology (Shandong Academy of Sciences), Jinan, China; ^6^ Qilu Hospital, Cheeloo College of Medicine, Shandong University, Jinan, China; ^7^ Drug Discovery Biology, Monash Institute of Pharmaceutical Sciences, Monash University, Melbourne, VIC, Australia

**Keywords:** atopic dermatitis, huangbai liniment (HB), CD4 T cells, regulatory T (treg) cell, immunomodulation

## Abstract

Atopic dermatitis (AD), also known as atopic eczema, is one of the most common skin diseases and is characterized by allergic skin inflammation, redness, and itchiness and is associated with a hyperactivated type 2 immune response. The leading causes of AD include an imbalance in the immune system, genetic predisposition, or environmental factors, making the development of effective pharmacotherapies complex. Steroids are widely used to treat AD; however, they provide limited efficacy in the long term and can lead to adverse effects. Thus, novel treatments that offer durable efficacy and fewer side effects are urgently needed. Here, we investigated the therapeutic potential of Huangbai Liniment (HB), a traditional Chinese medicine, using an experimental AD mouse model, following our clinical observations of AD patients. In both AD patient and the mouse disease model, HB significantly improved the disease condition. Specifically, patients who received HB treatment on local skin lesions (3–4 times/day) showed improved resolution of inflammation. Using the 1-Chloro-2,4-dinitrobenzene (DNCB)-induced AD model in BALB/c mice, we observed that HB profoundly alleviated severe skin inflammation and relieved the itching. The dermatopathological results showed markedly reversed skin inflammation with decreased epidermal thickness and overall cellularity. Correspondingly, HB treatment largely decreased the mRNA expression of proinflammatory cytokines, including IL-1β, TNF-α, IL-17, IL-4, and IL-13, associated with declined gene expression of IL-33, ST2, and GATA3, which are connected to the type 2 immune response. In addition, HB restored immune tolerance by promoting regulatory T (T_REG_) cells and inhibiting the generation of T_H_1, T_H_2, and T_H_17 cells *in vitro* and in the DNCB-induced AD mouse model. For the first time, we demonstrate that HB markedly mitigates skin inflammation in AD patients and the DNCB-induced AD mouse model by reinvigorating the T cell immune balance, shedding light on the future development and application of novel HB-based therapeutics for AD.

## Introduction

As the physical barrier of the host, the skin is the organ where dynamic environmental-host interactions occur to drive the host’s defense against stimuli, including microbial antigens and environmental chemicals, while abnormities in this response can lead to atopic reactions (Weidinger and Novak, 2016). Atopic dermatitis (AD), also known as atopic eczema, is a chronic allergic inflammatory skin disease that affects millions of people worldwide. Patients with AD exhibit mild to severe symptoms and often display eczematous lesions, erythema, pruritus, and skin inflammation (Leung et al., 2004; Weidinger and Novak, 2016). A proportion of AD patients also show comorbidities, including food allergies, asthma, allergic rhinitis, and other immune-mediated inflammatory diseases (Weidinger and Novak, 2016). A key pathophysiological mechanism of AD is the inappropriate immune response to antigens in the skin, which results in abnormalities of the epidermal structure and function and serious skin inflammation (Leung et al., 2004; Biedermann et al., 2015; Weidinger and Novak, 2016). Immune cells largely contribute to the development and regulation of AD, where the imbalance of CD4 T helper cell subsets is considered to be one of the major causes of the disease (Biedermann et al., 2015). Therefore, targeting the immune system imbalance is a promising approach to treat AD patients (Leung et al., 2004; Kim et al., 2019).

CD4 T helper cells play a vital role in the pathogenesis of AD. Emerging studies have shown that increased frequencies of T_H_1, T_H_2, T_H_17, and T_H_22 together with an excessive accumulation of inflammatory cytokines indispensably contribute to the onset of AD (Grewe et al., 1998; Weidinger and Novak, 2016). Specifically, the type 2 immune response and enhanced IgE response to allergens are the major factors that induce AD (Grewe et al., 1998; Weidinger and Novak, 2016). Antigen-primed T_H_2 cells and their associated cytokines, including IL-4, IL-5, and IL-13 induce immune dysfunction and subsequently damage cutaneous tissue integrity, leading to AD initiation (Suárez-Fariñas et al., 2011; Dainichi et al., 2018). In chronic lesions of AD, both T_H_2 and T_H_1 cells induce further cellular infiltration, while T_H_17 and T_H_22 cells play important roles in acute lesions (Nograles et al., 2009; Gittler et al., 2012). It is thus critical to inhibit the excess of these pro-AD T cell subsets to improve AD in patients. Regulatory T (T_REG_) cells regulate inflammation by substantially inhibiting proinflammatory T cells (Lei et al., 2015). By producing IL-10 and TGF-β, T_REG_ cells induce immune tolerance suppressing the severity of AD (Biedermann et al., 2015). Moreover, T_REG_ effector memory cells are reported to display the highest suppressive function (Rostaher et al., 2018; Looman et al., 2020). Furthermore, T_H_2-cytokine-producing type 2 innate lymphoid cells are also found in AD lesions and contribute to IL-17 dependent inflammation (Imai et al., 2013).

Although significant progress has been made in understanding AD pathogenesis, the lack of durable and well-tolerated treatments has led to significant health and economic burdens in managing AD in patients. Immunosuppressive drugs, such as steroids, are most commonly used to treat AD; however, side effects include severe immunosuppression, increased risk of opportunistic infection, and osteoporosis (Nguyen et al., 2019). Thus, novel therapeutics that can provide long-term benefits and are well tolerated in AD patients are urgently needed.

Emerging studies have demonstrated that traditional Chinese medicine provides desirable therapeutic effects in inflammatory diseases. Huangbai Liniment (HB) is a standardized medicinal product clinically prescribed for use in dermatology and wound management (Liu et al., 2020; Zhang et al., 2020). It is a compound traditional Chinese medicine that includes forsythia, honeysuckle, phellodendron, dandelion, and centipede. In clinical trials, HB has been reported to exert profound anti-inflammatory effects during wound healing in several chronic inflammatory diseases, such as diabetic foot ulcers (Liu et al., 2020; Zhang et al., 2020), ulcerative colitis (Xiao et al., 2009), and traumatic infection (Li-yun, 2016). Mechanistically, experimental and clinical data indicate that HB accelerates diabetic wound healing via activation of Nrf2 and its downstream antioxidant genes (Zhang et al., 2020). Although the protective effects of HB are reported in many mucosal damage and inflammatory diseases in clinical therapy, the therapeutic effects and mechanism of HB in ameliorating dermatitis remains unclear. In this study, we investigated the therapeutic potential of HB in controlling inflammatory dermatoses in a DNCB-induced AD mouse model following clinical observations in AD patients and found that HB markedly alleviated skin inflammation and reinvigorated T_REG_-induced immune balance in AD.

## Materials and Methods

### Patient

Using the Hanifin and Rajka AD diagnostic guidelines and the eczema area and severity index (EASI) AD guidelines as standard, patient was selected from the diagnostic clinic of Dongzhimen Hospital for AD diagnosis. AD patient had no history or family history of allergic disease. Patient had not received steroid or immunosuppressive therapy and had no history of parasitic infection 2 weeks before the examination. The study design was approved by the Ethics committee of Beijing University of Chinese Medicine (NO. ECSL-BDY-2012-45-01). Written informed consent was obtained from patient included in the study.

### Animals

All experimental 6–8 week-old BALB/c female mice were purchased from Vital River Laboratories (VRL, Beijing, China), and maintained at the Shandong Analysis and Test Center at a controlled temperature of 23 °C ± 2°C, and a humidity of 50 ± 10%. All animal work was in accordance with protocols approved by the Animal Experimentation Ethics committee of Shandong Analysis and Test Center (NO. ECAESDATC-2018-010) and followed the ARRIVE (Animal Research: Reporting of *In Vivo* Experiments) guidelines.

### Reagents

The HB (lot number: 18010111) used in this study was supplied by Shandong Hanfang Pharmaceutical Co., Ltd (Chinese medicine character: Z10950097). Hydrocortisone butyrate cream and 2, 4-Dinitrochlorobenzene (DNCB) were purchased from Jinyao Medical & Pharmaceutical Company Ltd. and Shanghai Chemical Reagent Company Ltd., respectively. The mouse antibodies against specific antigens used in flow cytometry analysis were purchased from BD Biosciences (San Jose, CA, United States) or BioLegend (San Diego, CA, United States). Recombinant murine IL-1β, IL-2, IL-4, IL-6, TGF-β, and IL-12 were purchased from PeproTech (Rocky Hill, NJ, United States). All other reagents were purchased from Sigma-Aldrich (St. Louis, MO, United States).

### Experimental 1-Chloro-2,4-Dinitrobenzene-Induced AD Mouse Model

For induction of AD-like skin lesions in an experimental mouse model, mice were topically sensitized with 100 μl of 1% DNCB, diluted in a 4:1 mixture of acetone and olive oil, on shaved dorsal skin three times a week. From day five, mice were challenged with 0.4% DNCB on the dorsal skin (100 μl) and right ear (10 μl) every 3 days. The HB or hydrocortisone was applied by hydropathic compress on treatment group mice twice a day for 20 days (400 μl/day), and the control animals received the same volume of phosphate-buffered saline (PBS). At the end of the model construction, ear thickness measurements were performed with dial calipers as previously described (KimKim et al., 2013; Kim et al., 2014). For behavioral assessments, mice were acclimated to the recording cage and then evaluated for their scratching behavior. Total numbers of scratching bout per 10 min were record manually by an assessor who was blind to the experimental design. One scratching bout was defined as one instance of lifting the forepaw from the floor, scratching, and returning the paw to the floor or placing the paw around the animal’s mouth (Oetjen et al., 2017). The severity of dermatitis was evaluated according to symptoms, including scaling, erythema, erosion, and edema. Each symptom was scored as 0 (no symptom), 1 (mild), 2 (moderate), or 3 (severe). After 20 days, at the end of the DNCB treatment, murine ear skin tissues and dorsal skin tissues were fixed in 4% paraformaldehyde (PFA) and subjected to histopathological analysis and other related experiments. Representative data are from one of three independent experiments (*n* ≥ 5 in each group).

### Skin Histology

Dorsal skin tissues were fully fixed with 4% paraformaldehyde at the endpoint before embedding in paraffin, sectioning, and staining with hematoxylin and eosin (H&E). Morphological evaluation of inflammatory infiltrates, and structural alterations were determined at a magnification of ×200 or ×50. To score the skin histopathology, inflammatory cell infiltration, epidermal thickness, and hair follicles were assessed blind-based using a five-point grading system and evaluated by blind scoring from 1 to 5, resulting in an average pathology score for each section. The infiltrated cells and the thickness of the epidermis or dermis were measured. Five randomized fields were counted for each section slide and calculated from five animals. All histology results were measured by blind evaluation.

### Quantitative Real-Time Polymerase Chain Reaction

Total RNA was purified from skin tissue using TRIzol reagent (Invitrogen), and cDNA was synthesized from a total of 5 μg of RNA using RevertAid First Strand cDNA Synthesis Kit (Thermo Scientific) and amplified using qPCR according to the manufacturer’s instructions. All qPCRs were run on the Roche real-time system using SYBR Green Master Mix (Roche) and normalized with *β*-Actin. The sequences of the primers used for qPCR are shown in Table 1.

**TABLE 1:** Sequences of qPCR primers.

Gene	Forward (5′-3′)	Reverse (5′-3′)	Size
IL-1β	TGC​CAC​CTT​TTG​ACA​GTG​ATG	ATG​TGC​TGC​TGC​GAG​ATT​TG	136 bp
IL-4	TCA​ACC​CCC​AGC​TAG​TTG​TC	TCT​GTG​GTG​TTC​TTC​GTT​GC	184 bp
IL-10	CCA​GTA​CAG​CCG​GGA​AGA​CA	CAG​CTG​GTC​CTT​TGT​TTG​AAA​GA	121 bp
IL-13	CAG​CAT​GGT​ATG​GAG​TGT​GG	AGG​CTG​GAG​ACC​GTA​GTG​G	153 bp
IL-33	CTA​CTG​CAT​GAG​ACT​CCG​TTC​TG	AGA​ATC​CCG​TGG​ATA​GGC​AGA​G	136 bp
IFN-γ	GCG​TCA​TTG​AAT​CAC​ACC​TG	TGA​GCT​CAT​TGA​ATG​CTT​GG	129 bp
TNF-α	GCT​GAG​CTC​AAA​CCC​TGG​TA	CGG​ACT​CCG​CAA​AGT​CTA​AG	118 bp
GATA3	CAG​CTC​TGG​ACT​CTT​CCC​AC	GTT​CAC​ACA​CTC​CCT​GCC​TT	112 bp
RORγt	TGA​GGC​CAT​TCA​GTA​TGT​GG	CTT​CCA​TTG​CTC​CTG​CTT​TC	104 bp
β-actin	TGA​CAG​GAT​GCA​GAA​GGA​GA	CGCTCAGGAGGAGCAATG	75 bp

### *In vitro* Polarization

Naïve CD4^+^ T cells were sorted from the spleens of healthy female 8 week-old BALB/c mice using a flow cytometer (BD FACS Aria III, BD Biosciences). The purity of the naïve CD4^+^ T cells was >98%. Sorted naïve CD4^+^ T cells (50,000 cells per well) were cultured in a 96-well culture plate pre-coated with anti-CD3 (5 μg/ml) and anti-CD28 (5 μg/ml) and polarized under T_H_1 (IL-2 20 ng/ml, anti-IL-4 10 μg/ml and IL-12 20 ng/ml), T_H_2 (IL-2 20 ng/ml, anti-IFN-γ 10 μg/ml, anti-IL-12 10 μg/ml, and IL-4 20 ng/ml), T_H_17 (IL-2 20 ng/ml, IL-6 50 ng/ml, TGF-β 1ng/ml, IL-1β 10 ng/ml, anti-IL-4 10 μg/ml, and anti-IFN-γ 10 μg/ml), or T_REG_ cell (IL-2 20 ng/ml and TGF-β 2 ng/ml) differentiation conditions with or without 1/1000 H B for 4 days.

### MTT Assay

Sorted naïve CD4^+^ T cells were cultured with 20 ng/ml IL-2 with or without 1/1000 HB for 2 days, MTT solution was then added, and the sorted cells were incubated in the dark. After a 6 h incubation, solubilization buffer (10% SDS in 0.01 M HCl) was added, and the cells were cultured overnight. Cell proliferation was measured at an OD value of 570 nm.

### Flow Cytometry

Mouse splenocytes were stained with Fc-receptor blocking antibodies (clone 2.4G2, 1:100 dilution, BD Biosciences) for 10 min on ice to block non-specific staining. For surface staining, cells were washed once with PBS containing 2% heat-inactivated fetal bovine serum (FBS, Gibco) and stained in an appropriately diluted antibody solution for 40 min at 4 C. Dead cells were excluded after staining with 7-amino-actinomycin D (7-AAD, BioLegend). For intracellular staining of cytokines, PMA and ionomycin stimulated cells were washed once after surface staining and permeabilized using Cytofix/Cytoperm (BD Biosciences) for 40 min on ice. The antibodies were diluted in Perm/Wash Buffer (BD), and stained in an appropriately diluted antibody solution for 1 h at 4 C. For intranuclear staining of transcription factors, cells were washed once after surface staining and permeabilized using Foxp3/Transcription Factor Staining Buffer Set (eBioscience) for 40 min on ice. The specific antibodies were diluted in Fixation/Permeabilization buffer (eBioscience) and incubated for 1 h at 4 C. Data were collected on a BD FACS Aria III flow cytometer (BD Biosciences) and analyzed using FlowJo (BD Biosciences) software. Antibody information is presented in Table 2.

**TABLE 2:** Antibodies for FACS staining of mouse cells.

Antibody	Parameter	Clone	Vendor
CD44	FITC	IM7	BD
RORγt	PE	Q31-378	BD
IFN-γ	PE-CF 594	XMG1.2	BD
GATA3	PE-CF 594	L50-823	BD
CD4	PE-Cy7	RM4-5	BD
Foxp3	Alexa Fluor^®^ 647	MF23	BD
IL-17A	Alexa Fluor^®^ 647	TC11-18H10.1	BioLegend
CD3	Alexa Fluor^®^ 700	17A2	BioLegend
CD45	APC-Cy7	30-F11	BD
CD45R/B220	APC-Cy7	RA3-6B2	BD
CD62L	BV421	MEL-14	BD
IL-4	BV421	11B11	BD
ST2	BV421	DIH9	BioLegend
Ki67	BV605	16A8	BioLegend
CD127	BV605	A7R34	BioLegend
CD8α	BV605	53–6.7	BD
CD25	BV650	3C7	BD
7-AAD	—	—	BioLegend

### Statistical Analysis

All data are expressed as Standard Error of the Mean (SEM) and are representative of three independent experiments. In the heatmap diagram, Z-score is measured in terms of standard deviations from the mean. The z-score is calculated by subtracting the population mean from the raw score, then dividing the difference by the population standard deviation. Groups were compared using unpaired Student’s *t*-tests and one-way analysis of variance (ANOVA). Differences were considered to be statistically significant at *p* < 0.05.

## Results

### Huangbai Liniment Ameliorates Dermatitis in AD Patient and a 1-Chloro-2,4-Dinitrobenzene-Induced AD Mouse Model

**FIGURE 1:**
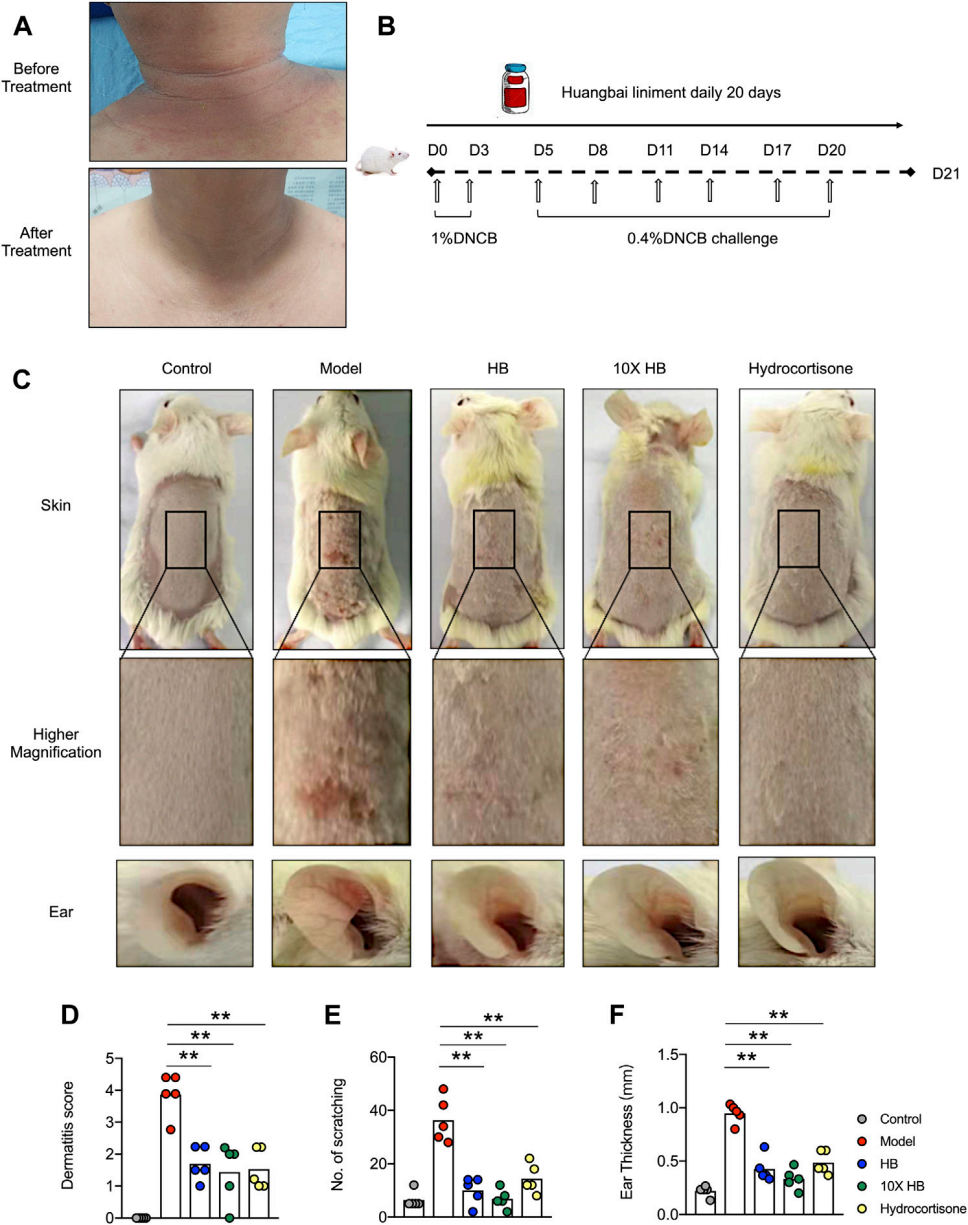
HB ameliorates the symptoms of AD in clinical AD patient and mice. **(A)** HB was topically applied to a patient with AD 3 times a day for a continuous 15 days. Representative image of dermatitis on the patient before and after treatment with HB. After HB application, the eczema on the skin of the AD patient’s neck was obviously ameliorated and the skin condition altered. **(B)** Schematic for (C–F). Mice were sensitized with 1% DNCB on dorsal skin at days 0 and 3 and then challenged with 0.4% DNCB on the dorsum of ears at 3 day intervals from days 5 to 20. HB, 10X HB (a 10-fold concentration of HB), hydrocortisone, or PBS was topically applied daily to the dorsal skin of the DNCB-induced mice. **(C)** Representative images of dorsal skins and ears in each group. The severity of AD was evaluated, and statistics are shown in **(D)**. **(E,F)** The amount of scratching and ear thicknesses were recorded at the endpoint, and statistics are shown. Each dot represents one mouse from one of three independent experiments, and bars indicate mean values. Statistical significance was determined by one-way ANOVA, *p < 0.05, **p < 0.01.

HB is a common dermatological medicinal product clinically prescribed to treat allergic contact dermatitis and eczema (Li and Gong, 2014; Yao and Zhao, 2014); however, its mechanism remains unclear. To evaluate the clinical efficacy and safety of HB in the treatment of AD, we locally applied hydropathic compresses of HB on lesioned skin of an AD patient. The AD patient presented with features of skin inflammation, including pruritus, erythema exudativum, and a mixed dermal inflammatory infiltrate around the head and neck region. The EASI score was around 13, and the lesion area was approximately 30–40% (Table 3). Several treatments had previously failed, and the patient had not received steroid or immunosuppressive therapy. The hydropathic compress of HB was administered to the lesioned skin region of the patient 3 times a day for 15 days. After the treatment, the AD patient showed marked improvement in AD clinical characteristics, including lessened random redness and limited itch symptoms (Figure 1A). These findings suggested that HB could be beneficial to patients with chronic skin inflammation who might be resistant to conventional treatments.

**TABLE 3:** Baseline and clinical characteristics of patient with AD.

Characteristics	Patient
Age (years)	52
Gender	Male
Disease severity	EASI score:13
Lesion Area	Head and neck, 30–40%
Signs and symptoms	
Eczema	Significant redness; score 3
Infiltration/papule	Infiltration occurred; score 3
Scales	Local mild desquamation, mainly fine scales; score 1
Exudation/crust	Extensive dermal edema; score 3
Pruritus	Frequent itching and scratching, affecting life and sleep, unbearable; score 3
Erosion	None; score 0
Family history	None

To investigate the underlying mechanism of HB on AD, a DNCB-induced AD mouse model was used (Figure 1B). Following DNCB treatment, the ears of the mice became red and swollen, and the dorsal skin showed severe erosion, erythema, and dryness. A variety of skin inflammation parameters and pathology were compared at the endpoint of the DNCB-induced AD mouse model. Interestingly, we found these atopic skin lesions were markedly attenuated by HB and 10-fold concentrated HB. The efficiency of HB was comparable with hydrocortisone, a common steroid medicine, which is widely used in the treatment of different dermatitis types (Figure 1C). Consistent with the photographic images of the skin lesions of the AD patient, the dorsal skin of the mice had obviously relieved edema, redness, and erosion following HB treatment (Figure 1D). HB also suppressed scratching behavior due to defused itching and skin lesions in the DNCB-induced AD mouse model group (Figure 1E, Supplementary Figure S1A) and ear thickness was also significantly thinner than in the DNCB-induced AD mouse model group (Figure 1F, Supplementary Figure S1B). Together, these results demonstrate that HB profoundly ameliorated the clinical symptoms of AD in mice.

### Huangbai Liniment Improves Symptoms of AD Through the Inhibition of Local Inflammatory Cytokines and Allergic Reactions

**FIGURE 2:**
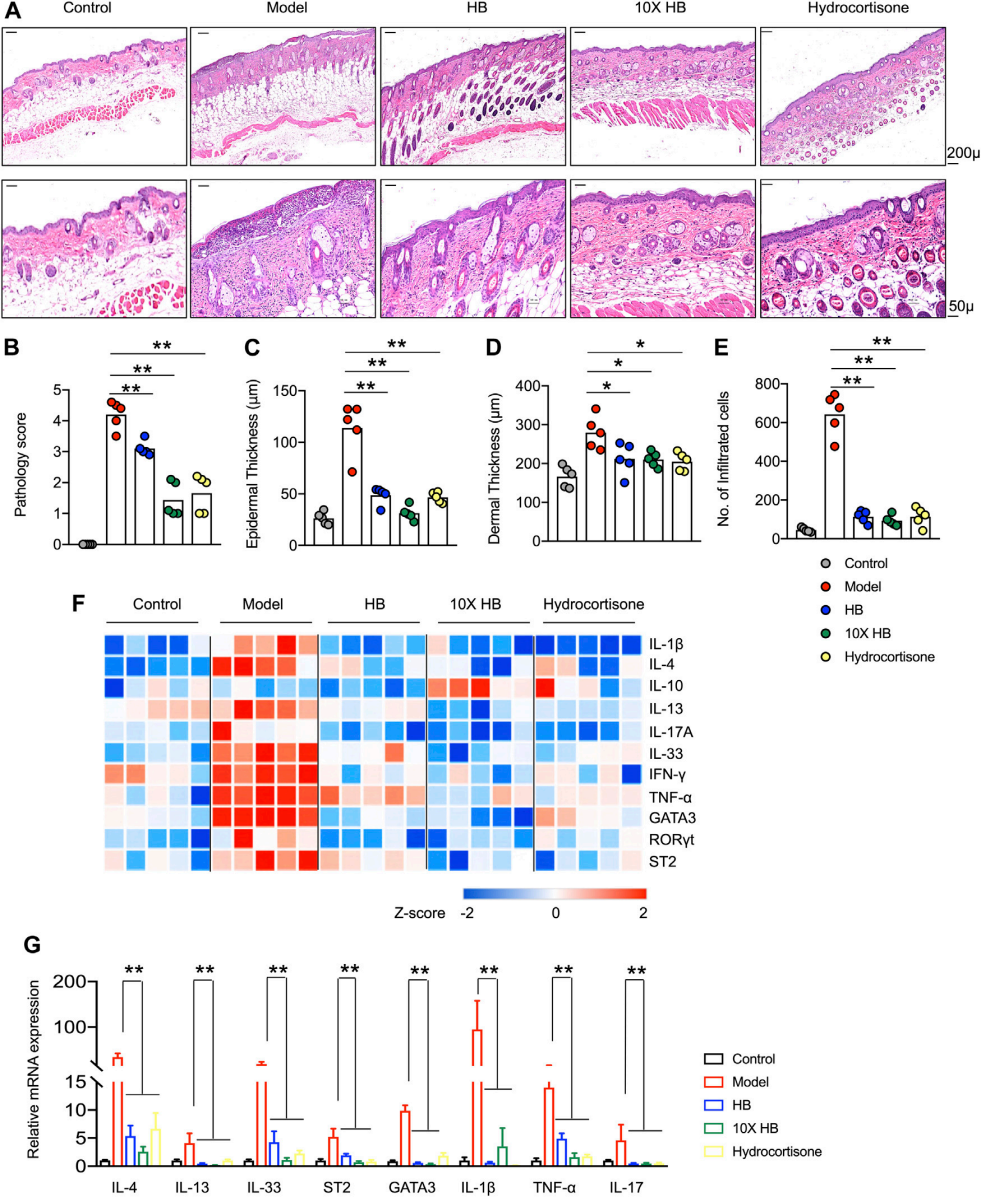
HB reduces AD-like skin inflammation and the type 2 immune response induced by DNCB in mice. Mice were sensitized with 1% DNCB on dorsal skin at days 0 and 3 and then challenged with 0.4% DNCB on the dorsum of both ears at 3 day intervals from days 5 to 20. HB, 10X HB (a 10-fold concentration of HB), hydrocortisone, or PBS was topically applied daily to the dorsal skin of these DNCB-induced mice. **(A)** Histopathology of mouse dorsal skin tissue was evaluated by H&E staining (scale bars, 50 or 200 μM). Representative images in each group are shown. Pathology scores were blindly evaluated, and statistics are shown in **(B)**. **(C–E)** ImagePro10 software was used to measure and calculate epidermal thickness **(C)**, dermal thickness **(D)**, and number of infiltrated cells **(E)**. **(F)** Gene expression in skin tissue from five individual mice in each group, measured by qPCR and shown in a heatmap. Blue represents low expression, and red represents high expression of each inflammatory gene compared between three groups, with Row Z-Score (−2–2). **(G)** Type 2 cytokines and transcription factors in skin tissues were measured by qPCR. Each dot represents one mouse from one of three independent experiments, and bars indicate mean values. Statistical significance was determined by one-way ANOVA, *p < 0.05, **p < 0.01.

The improvements in the atopic symptoms of mice in the DNCB-induced AD mouse model group following HB and conventional hydrocortisone treatments were confirmed by morphological analysis of H&E-stained sections from damaged dorsal skin at the endpoint (Figure 2A). The DNCB-induced AD mouse model group exhibited the typical microscopic characteristics of dermatitis, such as hyperkeratosis, thickened epidermal tissue, and immune cell infiltration. However, in skin tissue from HB and 10-fold concentrated HB treated mice, the epidermal and dermal tissues were markedly thinner than tissue from untreated mice. Furthermore, the infiltration of inflammatory cells was also significantly reduced by HB treatment (Figures 2B–E). Accordingly, the pathology score, mRNA expression of inflammation, and the type 2 cytokine-related genes, including IL-1β, TNF-α, IL-17A, IFN-γ, IL-4, IL-13, and IL-33, were largely downregulated by HB treatment. In contrast, HB increased the expression of anti-inflammatory cytokine IL-10 (Figure 2F). Moreover, the mRNA expression of T_H_2 and T_H_17 classical transcription factors, GATA3 and RORγt, were suppressed in HB-treated mice compared with untreated mice (Figures 2F,G). Collectively, these data suggest that HB improves AD and largely rescues the excessive expression of cytokines critical for skin inflammation and the atopic response.

### Huangbai Liniment Relieves Skin Inflammation by Modulating the Immune Balance of T_H_17/T_REG_ Cells

**FIGURE 3:**
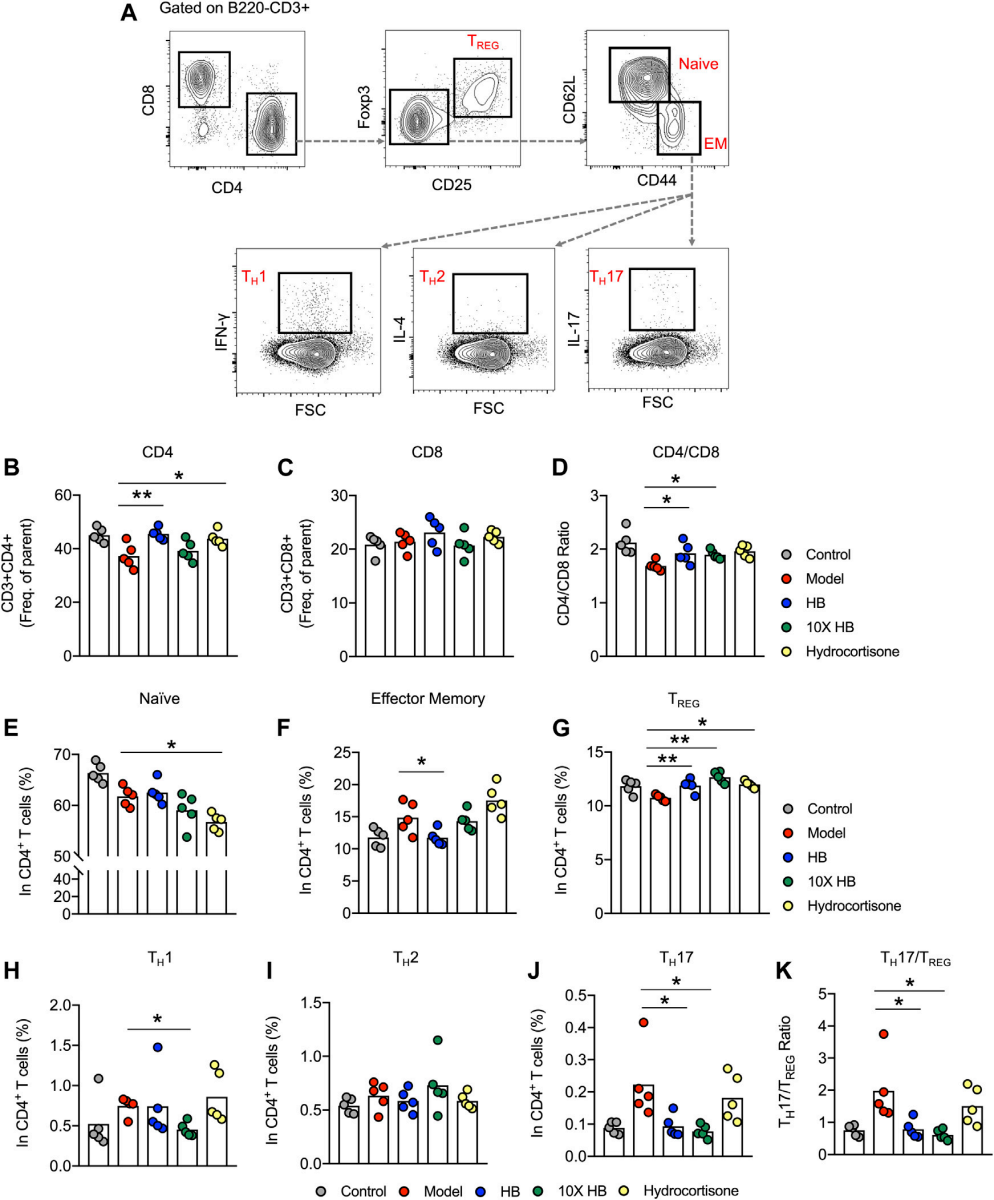
HB relieves skin inflammation by downregulation of the T_H_17/T_REG_ ratio. Mice were sensitized with 1% DNCB on dorsal skin at days 0 and 3, and then challenged with 0.4% DNCB on the dorsum of both ears at 3 day intervals from days 5 to 20. HB, 10X HB (a 10-fold concentration of HB), hydrocortisone, or PBS, was topically applied daily to the dorsal skin of the DNCB-induced mice. Splenocytes of AD model mice treated with PBS, HB, 10X HB, and hydrocortisone were isolated and stained using immunofluorescence antibodies. **(A)** Gating strategy for different T cell subsets. **(B,C)** Statistical analysis of the frequencies of B220^−^CD3^+^CD4^+^ T cells **(B)** and B220^−^CD3^+^CD8^+^ T cells **(C)** in different groups. **(D)** CD4/CD8 ratio. **(E–J)** Statistical analysis showing the differences in the frequencies of naïve **(E)** and effector memory **(F)** CD4 T cells and T_REG_ **(G)**, T_H_1 **(H)**, T_H_2 **(I)**, and T_H_17 **(J)** cells. **(K)** The T_H_17/T_REG_ ratio was calculated to show the differences in different groups. Each dot represents one mouse from one of three independent experiments, and bars indicate mean values. Statistical significance was determined by one-way ANOVA, *p < 0.05, **p < 0.01.

To clarify the underlying mechanism of HB-based immunomodulation, we analyzed mouse splenocytes in different treatment groups using multi-color flow cytometry to characterize the phenotype of CD4^+^ T cell subsets in DNCB-induced skin inflammation. We established gating strategies based on the key cytokines produced by each cell type to evaluate the different CD4^+^ T cell subsets (Figure 3A). Intriguingly, we found altered frequencies of CD4^+^ T cells in the different groups. Specifically, HB treatment reduced the frequency of CD4^+^ T cells that were substantially reduced in the DNCB-induced AD mouse model group (Figure 3B), while no difference was observed in the frequencies of CD8^+^ T cells (Figure 3C), which led to an increased CD4/CD8 ratio in the DNCB-induced AD mouse model group after HB treatment (Figure 3D). Furthermore, using the gating strategy of naïve (CD25^−^CD62L^+^CD44low), effector memory (CD25^−^CD62LlowCD44^+^), T_REG_ cells (CD25^+^Foxp3^+^) CD4^+^T cells (Figure 3A), we noted a comparable frequency of naïve CD4 T cells (Figure 3E), but a significant reduction in effector memory CD4 T cells in HB-treated mice compared with untreated mice (Figure 3F). T_REG_ cells have been shown to control excessive T cell responses in inflammation, atopic responses, and AD-like inflammation (Ou et al., 2004; Fyhrquist et al., 2012). Interestingly, we also noticed that HB increased the frequency of T_REG_ cells in the DNCB-induced AD mouse model group, which partially constituted the underlying mechanisms of HB-induced improvement of skin inflammation in our DNCB-induced AD mouse model (Figure 3H).

Effector CD4^+^ T cells are known to contribute to the pathology of AD. T_H_1, T_H_2, and T_H_17 are examined by gating on CD25^−^CD4^+^CD44^+^ T cells and through the expression of their signature cytokines (T_H_1: IFN-γ^+^CD44^+^; T_H_2: IL-4^+^CD44^+^; T_H_17: IL-17A^+^CD44^+^). We found that HB treatment reduced the frequency of T_H_17 and slightly suppressed the T_H_1 subset in the DNCB-induced AD mouse model group (Figures 3I,K). Comparable frequencies were observed for T_H_2 cells in untreated and HB-treated mice (Figure 3J). T_H_17/T_REG_ imbalance is widely reported in autoimmune, inflammatory, and allergic diseases (Ma et al., 2014). We next analyzed the percentage of T_H_17 and T_REG_ cells in different mouse groups. The ratio of T_H_17/T_REG_ was compared between untreated and HB-treated mice, and a significant reduction in the ratio was noticed in HB-treated mice (Figure 3L). Taken together, we reveal that HB treatment plays an important role in controlling AD-like inflammation by potentiating T_REG_ cells while reducing T_H_17 cells *in vivo*.

### Huangbai Liniment Directly Promotes T_REG_ Cells and Inhibits Effector T Cell Subsets *in vitro*

**FIGURE 4:**
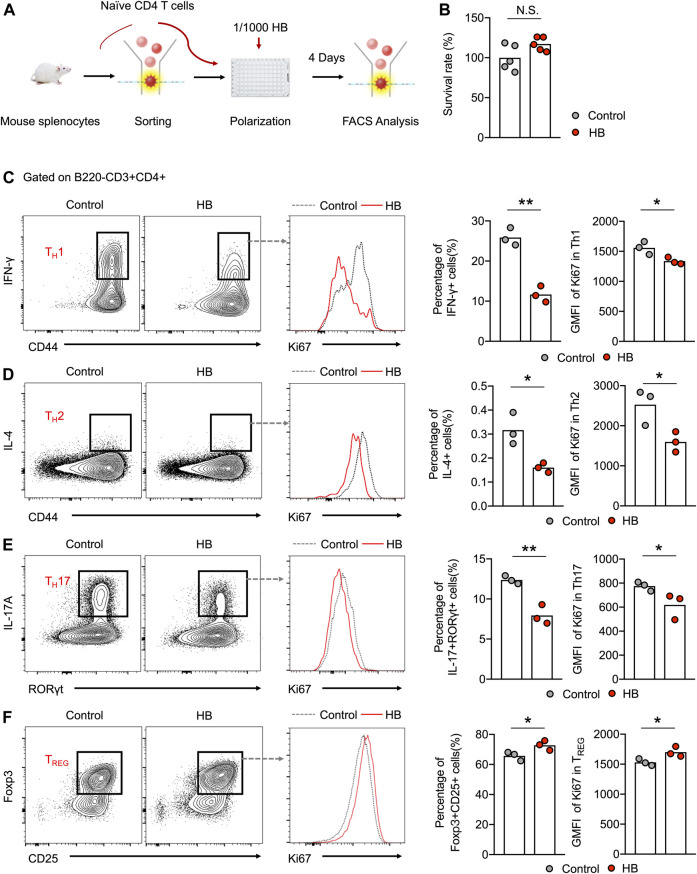
HB directly potentiates regulatory T cell activation and depresses inflammatory cells *in vitro*. **(A)** Schematic *in vitro* experiments. Naïve CD4 T cells, derived from mouse splenocytes, were sorted by flow cytometry and polarized under different culture conditions with or without 1/1000 HB for 4 days. **(B)** Survival analysis of HB for naïve CD4 cells was performed using an MTT assay after 48 h of culture with or without 1/1000 H B. **(C–F)** Representative FACS plots and statistical analysis of *in vitro* polarized T_H_1 **(C)**, T_H_2 **(D)**, T_H_17 **(E)**, and T_REG_ **(F)** cells with or without HB treatment. Histogram plots and statistical analysis indicated the changes in Ki67 in different CD4 subsets after HB treatment. Each dot represents one mouse from one of three independent experiments, and bars indicate mean values. Statistical significance was determined by Student’s *t*-test, *p < 0.05, **p < 0.01.

To further evaluate whether HB can directly affect the differentiation of different T cell subsets, we *in vitro* polarized purified naïve CD4^+^ T cells into T_H_1, T_H_2, T_H_17, and T_REG_ cells and added HB to the cell culture (Figure 4A). Based on the cell survival data, HB did not affect the survival of naive CD4^+^ T cells when cultured in T_H_0 conditions for 48 h (Figure 4B). In line with the *in vivo* data, we found that HB directly suppressed the generation of T_H_1, T_H_2, and T_H_17 cells and promoted T_REG_ differentiation in cell culture with altered cell proliferation indicated by Ki67 (Figures 4C–F). This mechanistic evidence indicates that HB directly promotes T_REG_ cells while largely inhibiting the generation of T_H_1 and T_H_17 cells.

## Discussion

AD is a chronic relapsing inflammatory skin disease characterized by an impaired immune response (Auriemma et al., 2013). The relapsing and persistent itch triggers a self-perpetuating itch-scratch cycle, which can have a significant impact on the patient’s quality of life, and effective treatments are limited (Oetjen et al., 2017). HB was reported to have therapeutic effects on different types of inflammation in patients, especially in those patients with eczema (Li and Gong, 2014; Yao and Zhao, 2014; Li-yun, 2016). However, it is unknown whether HB could be used to treat skin inflammation, such as AD, and if so, what are the underlying mechanisms of HB-mediated anti-inflammatory effects. In this study, we first examined the therapeutic potential of HB in a patient with AD and found that HB profoundly mitigated skin inflammation in the patient. Further, we investigated the therapeutic effects of HB on DNCB-induced AD in mice and examined its immune-modulative effects on CD4^+^ T cell subsets. In mice with DNCB-induced AD, HB markedly alleviated skin inflammation and inhibited proinflammatory immune responses mediated by Th1 and Th17 cells. This evidence suggests that HB effectively mitigates skin inflammation in mice with DNCB-induced AD by restoring the balance of the CD4^+^ T cell response. Our study also provides insights for the future development and application of HB-based immune-modulation of AD.

Type 2 cytokines, such as IL-4, IL-5, and IL-13 coordinate inflammatory and atopic responses in AD (Bieber, 2010). In our study, we found that HB markedly suppressed mRNA expression of IL-4, IL-13, and the transcription factor GATA3 known to drive immune cells to produce a type 2 immune response in dermatitis skin tissue. IL-33 and its receptor ST2 are strongly associated with the development of AD disease triggered by allergen exposure, irritants, scratching, and the bacterial and viral infections seen in this condition (Savinko et al., 2012). We noticed that that HB inhibits DNCB-induced IL-33 and ST2 mRNA levels in dermatitis skin tissue. Because ST2 is also expressed on innate lymphoid cells, such as ILC2 cells that are known to synergize with T_H_2 cells to induce type 2 inflammation (Artis and Spits, 2015; Eberl et al., 2015; Zhou et al., 2021), it will be interesting in future studies to know whether HB could directly regulate ILC2s to control skin inflammation in AD mouse models and patients. IFN-γ mRNA and protein were highly expressed in eczematous skin in the vast percentage of AD patients (Leung et al., 2011; Weidinger and Novak, 2016). More importantly, the *in situ* expression of IFN-γ decreased with the successful treatment of AD patients (Grewe et al., 1998). We also found that HB could largely reduce the mRNA expression of IFN-γ in skin tissue from mice with DNCB-induced AD. Together, our data indicate that HB suppresses the overall production of inflammatory cytokines in the skin of mice with DNCB-induced AD-like inflammation.

An imbalanced immune response is one of the major causes of AD. An excessive accumulation of activated T lymphocytes in the skin drives severe inflammation and induces destruction of the skin tissue integrity. Several effector subsets of CD4 T cells have been identified as potent regulators of AD pathogenesis, including T_H_1, T_H_2, T_H_17, and T_REG_ cells (Grewe et al., 1998; Martel et al., 2016). We found that HB treatment restored the CD4/CD8 T cell ratio in mice with DNCB-induced AD-like inflammation. Our data also suggested that DNCB-induced AD-like inflammation in mice was associated with reduced naïve cells and increased effector memory CD4^+^ T cells, and this imbalance was largely rescued after HB treatment. Activated T_H_2 cells prominently mediated the development of AD-like skin inflammation, manifesting as enhanced IgE-mediated sensitization and eosinophil infiltration (Bieber, 2010). Other effector T cell subsets, such as proinflammatory cells T_H_1, T_H_9, T_H_17, and T_H_22, produced a large number of proinflammatory cytokines, including IFN-γ, IL-9, IL-17, and IL-22, driving the development of AD. This promotion of effector CD4^+^ T cells was often associated with a decline in the presence of T_REG_ cells that are essential for immune tolerance and control of inflammation in AD (Auriemma et al., 2013). Our study further revealed that HB could potentiate T_REG_ cells while suppressing other effector CD4^+^ T cell subsets both *in vivo* and *in vitro*, which at least partially constituted the mechanisms of HB-mediated improvement in AD.

Th17 cells are critical for the development of AD (Koga et al., 2008). The frequency of IL-17-producing CD4^+^T cells in AD patients is increased and associated with AD severity (Esaki et al., 2016). In addition, IL-17 was reported to trigger the production of IL-4 by T_H_2 cells (Milovanovic et al., 2010). Mice lacking IL-17A exhibited reduced dermatitis together with less IL-4 and IgE production (Milovanovic et al., 2010). In line with these reports, our study showed that the frequency of Th17 cells was drastically reduced by HB *in vitro* and *in vivo*. The local expression of Th17-related cytokines and transcription factors were also shown to be suppressed by qPCR. These findings support the notion that HB directly regulates the differentiation of T_H_17 cells, could have immune-modulative effects in other inflammatory diseases and might be beneficial in controlling lesioned skin of sclerosis (Ahmed et al., 2019) or psoriasis, which are largely IL-17-driven diseases (Noda et al., 2015).

Interestingly, T_H_17 and T follicular helper (T_FH_) cells produce IL-21, which promotes T_H_17 cell differentiation (Ouyang et al., 2008; Gong et al., 2019), and IL-21 is increased in AD lesions in humans and mice (Jin et al., 2009). Moreover, IL-21 suppresses the production of IgE and possesses anti-inflammatory effects (Ozaki et al., 2002). The administration of IL-21 not only reduces the frequency of T_H_2 cells but also suppresses the secretion of T_H_2-associated cytokines, such as IL-4, IL-5, and IL-13 (Lin et al., 2015). Additionally, T_FH_ cells predominantly produce IL-2, IL-4 and IL-21 in B cell follicles and closely regulate antibody class-switching in severe inflammatory and allergic diseases, including AD, asthma, and COVID-19-induced airway inflammation (Yao et al., 2018; Crotty, 2019; Gong et al., 2019; Papillion et al., 2019; Yao et al., 2019; Zhou et al., 2019; Gong et al., 2020). Further, circulating T_FH_ cells are associated with disease severity in children with AD (Gong et al., 2019; Marschall et al., 2021). These accumulating findings indicate the importance of T_FH_ cells in the context of AD and other inflammatory diseases. It is thus of interest to know whether HB treatment also regulates T_FH_ cells in the context of AD in the DNCB-induced AD mouse model and patients.

In contrast to the reduction in proinflammatory CD4^+^ T cell subsets, HB enhanced T_REG_ cells *in vivo* and *in vitro*. In addition to the T_H_1/T_H_2 paradigm, the imbalance of T_REG_ and T_H_17 cells is another cardinal mechanism in AD pathogenesis (Ma et al., 2014). Our data importantly showed that HB potently restored this imbalance in mice with DNCB-induced AD. Future studies should further demonstrate the potential molecular insights underlying HB-mediated direct regulation of T_REG_ cells and other effector CD4^+^ T cell subsets, including but not limited to transcriptional regulation, epigenetic changes, and metabolic alterations.

In summary, our findings indicate that HB treatment improves AD in patients and can ameliorate skin inflammation in mice with DNCB-induced AD. HB profoundly reduced the expression of proinflammatory cytokines, including IL-1β, IL-4, IFN-γ, IL-13, and IL-17, and increased the expression of anti-inflammatory cytokine IL-10. Furthermore, we demonstrated that HB reduced skin inflammation and inhibited T_H_17 cells while promoting T_REG_ cells. In line with *in vivo* data, HB directly inhibited T_H_1, T_H_2, and T_H_17 differentiation and promoted T_REG_ polarization *in vitro*. Altogether, this study provides mechanistic insights into HB-mediated amelioration of AD through the restoration of CD4^+^ T cells and sheds light on the broad use of HB in treating inflammatory diseases.

## Data Availability

The raw data supporting the conclusions of this article will be made available by the authors, without undue reservation.
