# Supplementary material for: Huangbai Liniment Ameliorates Skin Inflammation in Atopic Dermatitis
Source: Front Pharmacol. 2021 Aug 31;12:726035. doi: 10.3389/fphar.2021.726035 (PMC8438128; doi:10.3389/fphar.2021.726035)
Supplement: Supplementary file 1 [file Image1.pdf]

Supplementary Figure1

A.

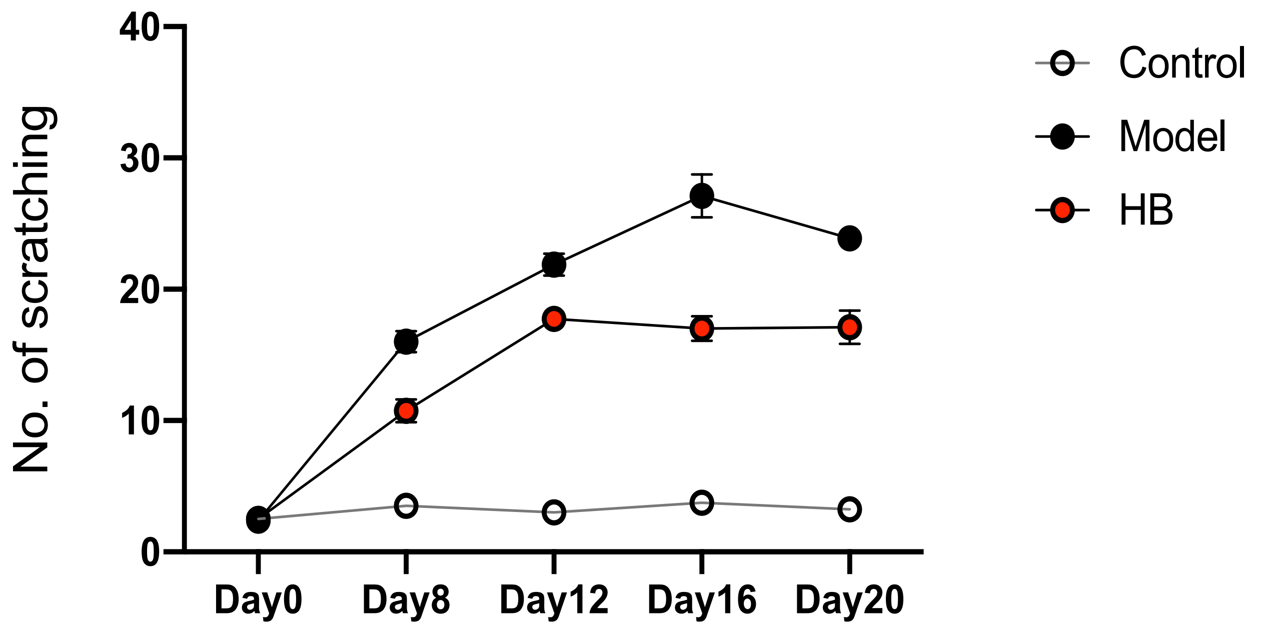

B.

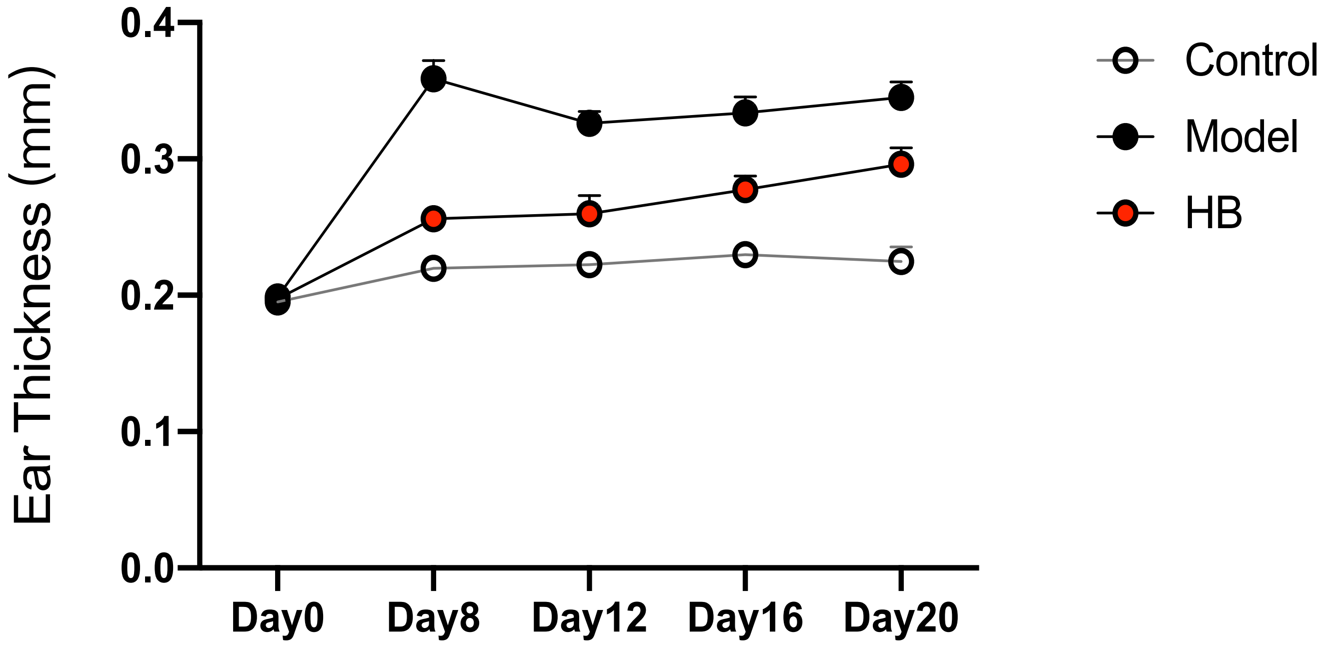

Supplementary Figure 1. The total number of scratching and ear thickness in mouse with DNCB induced atopic dermatitis. (A) Total number of scratching. (B) Ear thickness. Control N = 4, Model N = 8, HB N = 8. Error bar SEM.
